# Self-Resistance as a Functional Beacon: Target-Directed Microbial Genome Mining from Classical Discovery to Automated Pipelines

**DOI:** 10.3390/microorganisms14081762

**Published:** 2026-08-10

**Authors:** Jiaxin Wu, Mengxu Qiao, Yayue Ma, Jiaqi Liu, Jie Wei, Peng Zhang

**Affiliations:** 1 Laboratory of Microbial Resources and Synthetic Biology, School of Life Sciences, Inner Mongolia University, Hohhot 010010, China; 15174941718@163.com (J.W.); mengx_qiao@163.com (M.Q.); 13234809498@163.com (Y.M.); xiaoliu020814@163.com (J.L.); 2 Inner Mongolia Engineering Technology Research Center of Germplasm Resources Conservation and Utilization, Inner Mongolia University, Hohhot 010010, China

**Keywords:** self-resistance genes, genome mining, biosynthetic gene clusters (BGCs), target-directed discovery

## Abstract

Natural products remain a major source of structurally diverse and biologically active small molecules, yet traditional activity-guided discovery is labor-intensive and prone to rediscovery, while untargeted genome mining often lacks efficient prioritization criteria for biosynthetic gene clusters (BGCs). Self-resistance-gene guided discovery has emerged as a powerful strategy to address this limitation. In producing organisms, toxic metabolites are typically accompanied by genetically encoded self-protection mechanisms, such as resistant target homologs, duplicated housekeeping genes, detoxification enzymes, repair systems, or transporters. When co-localized with BGCs, these determinants serve as functional markers for predicting bioactivity and, in some cases, molecular targets prior to compound isolation. Over the past decade, this concept has evolved into a target-directed genome mining framework supported by tools and databases including ARTS, FunARTS, antiSMASH, and MIBiG. This review summarizes the biological basis, workflow, representative advances, and limitations of this strategy. Self-resistance genes can thus be viewed as functional beacons for accelerating bioactive natural product discovery.

## 1. Introduction

**Figure 1:**
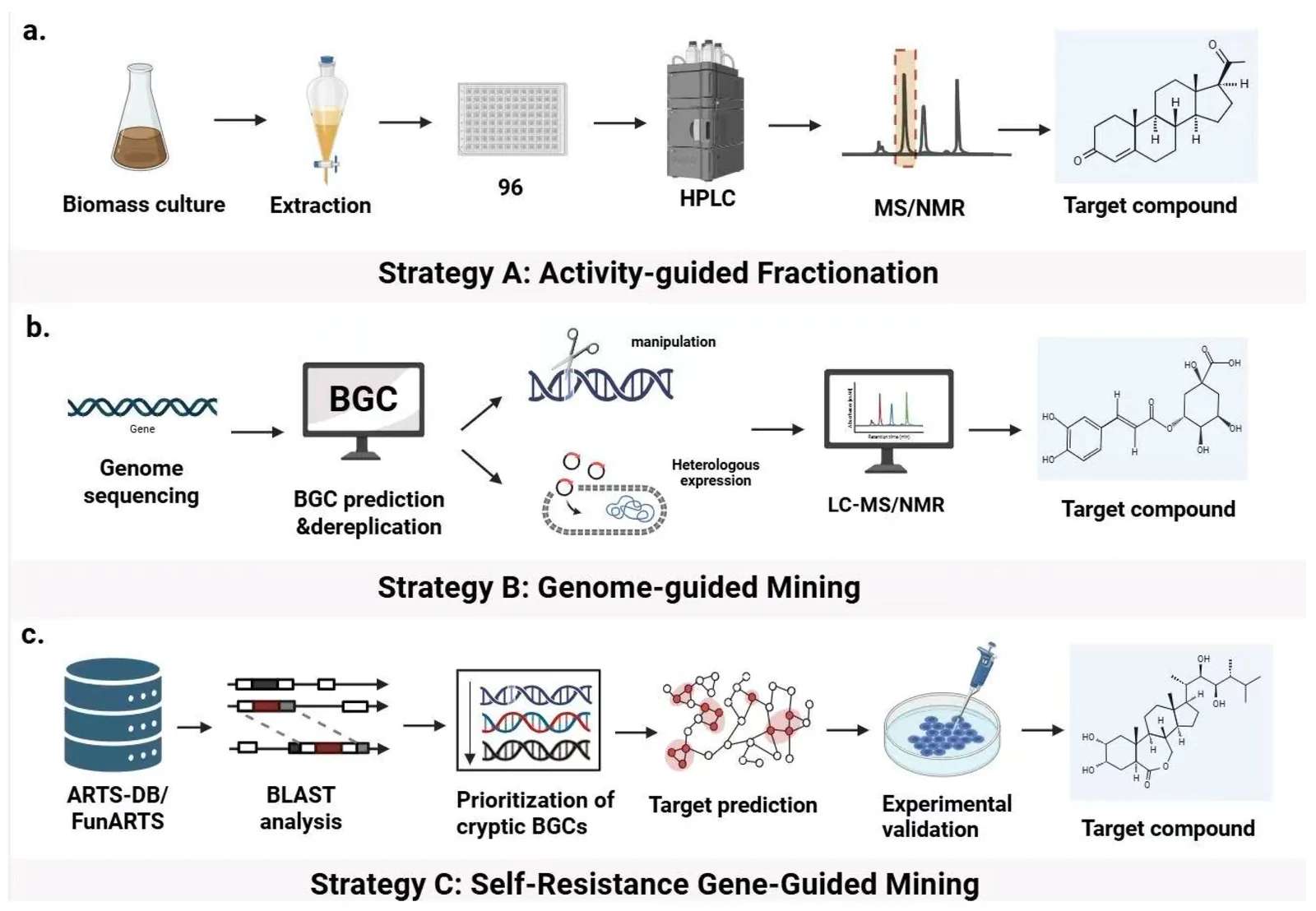
Schematic diagram of three microbial natural product discovery strategies. (**a**) Activity-guided fractionation isolates bioactive compounds via biomass culture, metabolite extraction, 96-well plate screening, HPLC separation, and MS/NMR structural characterization. (**b**) Genome-guided mining uses microbial genome sequencing and BGC prediction, with candidate BGCs validated via genetic manipulation, heterologous expression, and LC-MS/NMR analysis. (**c**) Self-resistance gene-guided mining employs ARTS-DB (https://arts-db.ziemertlab.com) /FunARTS (https://funarts.ziemertlab.com/, accessed on 1 August 2026) and BLAST (https://blast.ncbi.nlm.nih.gov/, accessed on 1 August 2026) for bioinformatic screening, prioritizes self-resistance gene-associated cryptic BGCs, and verifies candidate compounds through computational prediction and experimental assays to identify target natural products.

Microbial secondary metabolites exhibit diverse structural scaffolds and remarkable biological activities, making them an invaluable resource for novel drug discovery [1]. For decades, natural product discovery has relied primarily on activity-guided fractionation and conventional chemical screening (Figure 1a). However, these approaches are labor-intensive, time-consuming, and often lead to the repeated isolation of known compounds. With the rapid advancement of high-throughput genome sequencing technologies, it has become evident that microbial genomes encode far more biosynthetic gene clusters (BGCs) than the number of currently characterized natural products, suggesting that the majority of secondary metabolic pathways remain silent under standard laboratory conditions [2,3,4].

Genome mining has emerged as a powerful strategy to address these challenges by enabling the prediction of natural products directly from genetic information rather than observable phenotypes [5]. As illustrated in Figure 1b, the conventional genome mining workflow involves genome sequencing (e.g., Illumina/PacBio), followed by BGC identification and dereplication using bioinformatic tools and databases to prioritize cryptic or unknown clusters (Figure 1b). These candidate BGCs are then subjected to experimental validation through gene manipulation (e.g., knockout or silencing) or heterologous expression, coupled with metabolomic analysis and structural elucidation. Despite its potential, this untargeted approach is often limited by ambiguous correlations between BGCs and their corresponding metabolites, resulting in low efficiency in pathway activation and compound identification [6].

In recent years, self-resistance gene-guided genome mining has emerged as a more precise and efficient alternative (Figure 1c). This strategy is based on the observation that microorganisms producing toxic secondary metabolites typically harbor self-resistance genes within the same BGCs to avoid self-intoxication [7,8,9]. As shown in Figure 1c, self-resistance genes can be systematically identified using specialized tools and databases (e.g., ARTS 2.0, FunARTS (https://funarts.ziemertlab.com/, accessed on 1 August 2026), antiSMASH v8.0 [10,11,12,13], and MIBiG v4.0), followed by homology analysis to detect resistance elements associated with essential housekeeping enzymes. These genes act as functional markers that not only enable accurate prioritization of candidate BGCs but also allow direct inference of the molecular targets of the encoded natural products [14].

Conceptually, self-resistance strategy transforms BGCs from purely biosynthetic predictions into mechanistically informed hypotheses, focusing on whether gene clusters encode metabolites that interact with specific cellular targets or pathways—the core principle of target-directed genome mining [14]. Candidate self-resistance determinants are typically prioritized based on three key criteria: (i) duplication, referring to the presence of additional copies of essential or housekeeping genes that function as insensitive target homologs; (ii) proximity, indicating co-localization within or adjacent to BGCs, which strengthens functional association; and (iii) interpretability, meaning that candidate genes exhibit recognizable similarity to known targets or resistance elements, enabling mechanistic inference through sequence or structural features [15,16,17,18]. For example, the frzK gene associated with FR901483 biosynthesis encodes a resistant PPAT homolog that maintains purine biosynthesis, while β-lactam resistance genes are tightly organized within the cephamycin–clavulanic acid supercluster, and odilorhabdin resistance gene oatA is directly linked to its BGC [16,19,20].

Compared with conventional genome mining, this target-directed strategy significantly improves discovery efficiency by narrowing the search space and linking BGCs to their biological functions at an early stage. A major advantage lies in its ability to generate bioactivity and mode-of-action hypotheses prior to compound isolation, with resistance genes acting as ‘functional beacons’ that guide the identification of metabolites with defined targets [17]. Subsequent experimental validation—such as resistance phenotype assessment and target inhibition assays—can rapidly confirm both bioactivity and mechanism. This strategy has already enabled the elucidation of previously intractable pathways, such as cerulenin biosynthesis, and facilitated the discovery of diverse bioactive compounds including cytotoxic antibiotics, antitumor agents, and herbicides [16,21,22]. Not all resistance-associated genes represent true self-protection determinants, and many important BGCs lack obvious resistance markers, particularly when resistance relies on regulatory, physiological, or spatial mechanisms [18,23]. Emerging studies further suggest that self-resistance is not an isolated defensive process but is intricately integrated with biosynthetic pathways, co-evolving with metabolite assembly in a coordinated manner. The discovery of unconventional mechanisms—such as extracellular activation coupled with intracellular inactivation in fungal macrolides, or RTA1-like protein-mediated resistance in antifungal polyketides—has further expanded the conceptual framework of self-resistance [23,24].

A comprehensive review published in Natural Product Reports in 2020 summarized the progress of self-resistance-gene-guided discovery from 2000 to 2019, establishing a solid foundation for the field [14]. Since then, rapid advancements have been achieved, including the expansion to fungal genome mining, the identification of compounds targeting eukaryotic systems, the development of automated high-throughput platforms (e.g., FAST-NPS, originally developed for *Streptomyces*) for prokaryotic systems, and the integration of specialized computational tools and databases. Building upon this foundation, this review not only updates recent advances but also provides a systematic and critical evaluation of this strategy. Genome mining strategies integrating self-resistance gene analysis have unlocked novel bioactive metabolites from actinomycetes, fungi, and marine bacteria [25,26,27,28,29], with next-generation sequencing further expanding the discovery of cryptic biosynthetic clusters in underexplored microbes [30,31], thereby offering a timely and integrated framework for the field.

## 2. Molecular Mechanisms of Self-Resistance in Natural Product Producers

The biosynthesis of toxic natural products poses an inherent self-protection challenge for producing organisms, which have evolved four major categories of genetically encoded self-resistance mechanisms, with the most exploitable being those genetically linked to BGCs and mechanistically interpretable for target prediction [9,15,17].

### 2.1. Target Modification or Target Replacement

**Figure 2:**
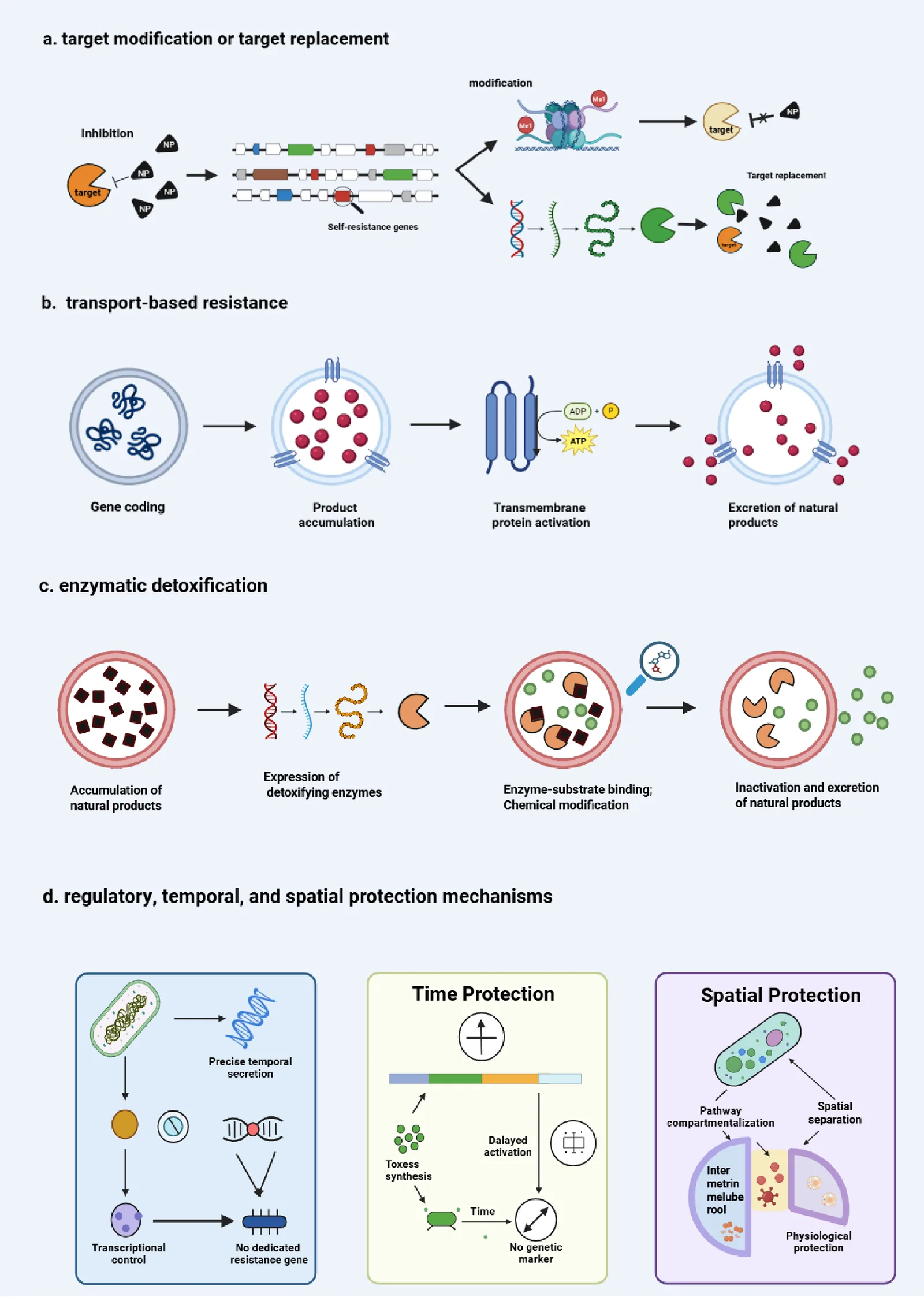
Mechanisms of self-resistance in natural product-producing microorganisms. (**a**) Target modification or replacement, where structural alteration or substitution of drug targets abrogates inhibitor binding; (**b**) transport-based resistance, involving transmembrane protein activation to drive efflux of endogenous bioactive metabolites; (**c**) enzymatic detoxification, through expression of detoxifying enzymes that chemically modify and inactivate toxic natural products; (**d**) Regulatory, Temporal, and Spatial Protection mechanisms, encompassing transcriptional/epigenetic regulation of resistance genes, temporal separation of toxin synthesis and target expression, and spatial compartmentalization of metabolites via organellar sequestration.

Target modification or replacement represents the most informative self-resistance mechanism for natural product discovery (Figure 2a). In this strategy, producing organisms encode resistant homologs of target proteins or duplicated housekeeping genes, allowing normal physiological function while reducing susceptibility to toxic metabolites [16,32]. These resistance determinants are typically co-localized with biosynthetic gene clusters (BGCs), providing direct and reliable clues for molecular target prediction [20,32].

Brief representative examples are listed below without detailed functional characterization (detailed mining cases are elaborated in Section 4): *Saccharothrix mutabilis* relies on phosphotransferase Cph for capreomycin self-protection [15]; fungal frzK confers resistance to the purine synthesis inhibitor FR901483 [16]. This mechanism underpins the discovery of cerulenin, DHAD-targeted herbicides and CYP51-binding antifungals via resistance-guided pipelines [18,22,32,33,34]. Collectively, target-homolog resistance genes serve robust predictive markers for metabolite bioactivity.

### 2.2. Transport-Based Resistance

Transport-based resistance is a widespread and versatile mechanism that protects producing organisms by reducing intracellular accumulation of toxic natural products through membrane transporters or efflux systems. Although these genes are useful for BGC identification, they generally provide less direct information about molecular targets compared to target-modifying resistance genes (Figure 2b).

Typical cases include fungal RTA1-type transporter AcrD in Acrophialophora levis [9], and coordinated efflux systems in *Streptomyces lusitanus* and *Pseudomonas fluorescens* that export inactive precursors for extracellular activation [23]. Fungal macrolide biosynthesis couples efflux with spatiotemporal control of toxicity [7]. Large-scale genomic surveys via Resistance Gene Database (RGDB) database confirm the wide distribution of transport resistance elements across microbes [17,35,36,37].

### 2.3. Enzymatic Detoxification or Damage Repair

Enzymatic detoxification enables producing organisms to inactivate toxic natural products through chemical modification or to repair molecular damage caused by these compounds (Figure 2c). Acetyltransferases are the most common detoxifying enzymes in bacteria, while DNA repair systems play a central role in producers of genotoxic metabolites [20,38,39,40].

Minimal cited examples: lasso peptide-modifying acetyltransferase in Paenibacillus sp., and OatA acetyltransferase inactivating odilorhabdin antibiotics [20]. Capreomycin producers combine acetyl- and phosphotransferase dual detoxification systems [15]. For genotoxic metabolites, DNA repair pathways act as core self-defense modules: cosmomycin D producers deploy mycothiol antioxidant systems, while myxin-synthesizing strains utilize monooxygenase detoxification [39,40]. Related resistance signatures also facilitate the identification of ClpP inhibitors and hybrid polyketide-peptide antimicrobials [21,38].

### 2.4. Regulatory, Temporal, and Spatial Protection Mechanisms

In addition to gene-encoded resistance factors, producing organisms employ regulatory, temporal, and spatial strategies to minimize self-toxicity (Figure 2d). These include delayed activation of natural products, compartmentalization of biosynthetic pathways, and precisely controlled secretion. Although biologically significant, these mechanisms are less amenable to large-scale computational mining due to the lack of distinct genetic markers [19,23,37].

Classic instances include extracellular precursor activation of kalimantacin and naphthyridinomycin [23], coordinated β-lactamase regulation in cephamycin-producing actinobacteria [19], and compartmentalized biosynthetic pathways for alligamycin and polyene macrolides [41,42]. Fungal macrolide pathways further integrate extracellular activation and intracellular detoxification cycles to avoid self-harm [7].

## 3. Computational and Experimental Workflow

Self-resistance gene-guided natural product discovery follows an integrated computational–experimental workflow that combines genome sequencing, bioinformatic prioritization, and functional validation to efficiently identify novel bioactive compounds. This pipeline transforms genomic data into mechanistically informed discovery routes, significantly improving both efficiency and success rate.

### 3.1. Genome Sequencing and BGC Annotation

**Figure 3:**
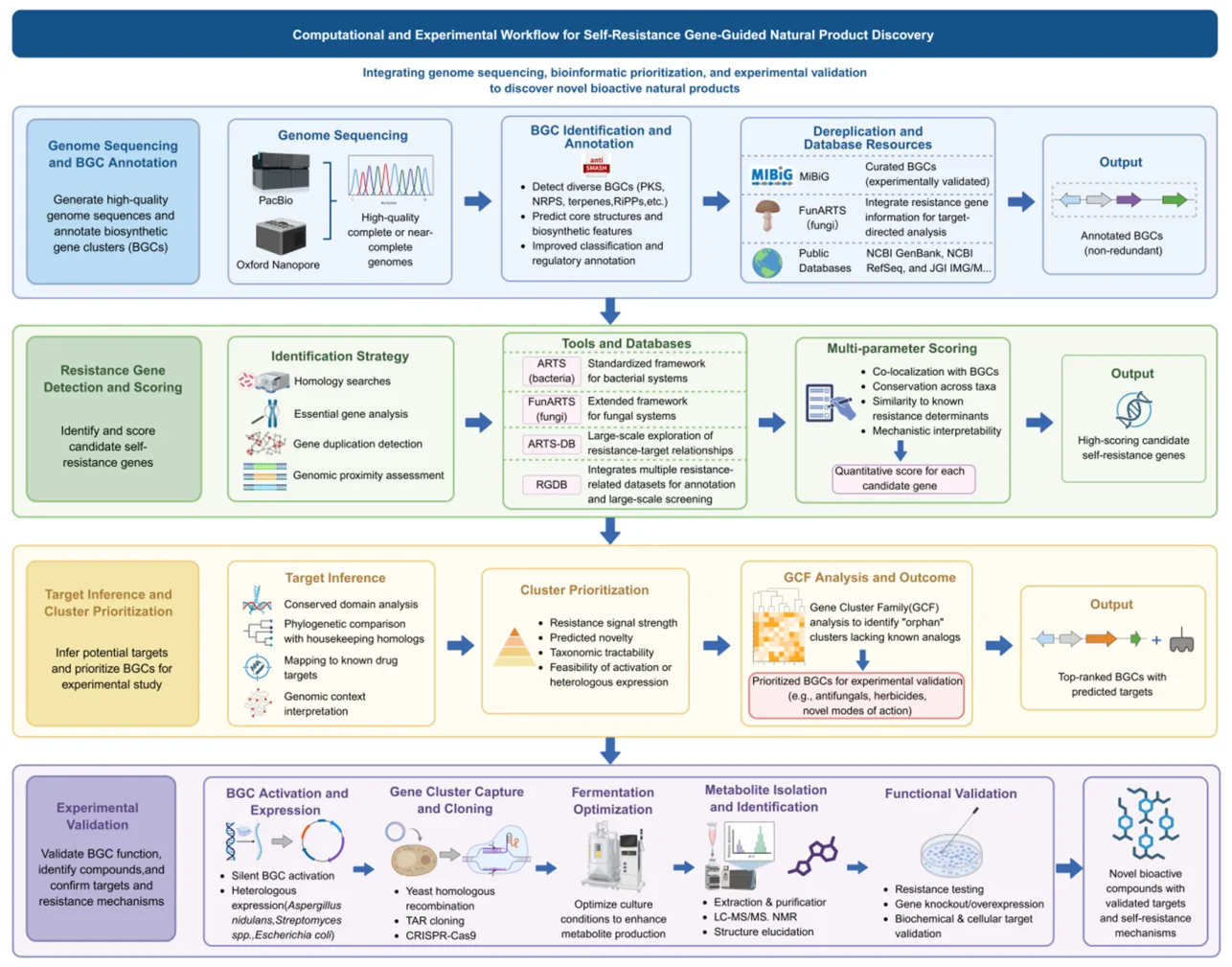
Workflow for Self-Resistance Gene-Guided Natural Product Discovery.

The workflow begins with the acquisition of high-quality genome sequences. Third-generation sequencing technologies, such as PacBio and Oxford Nanopore, now enable routine generation of complete or near-complete genomes, which is essential for preserving biosynthetic gene cluster (BGC) integrity and facilitating downstream functional studies (Figure 3). Subsequently, BGC identification and annotation are performed using bioinformatic tools. antiSMASH v8.0 remains the most widely used platform across bacteria, archaea, and fungi, enabling the detection of diverse BGC classes (e.g., PKS, NRPS, terpenes, and RiPPs) and providing predictions of core structures and biosynthetic features [13,43]. To avoid rediscovery of known compounds, dereplication is conducted using curated databases such as MIBiG v4.0(Minimum Information about a Biosynthetic Gene cluster), which contains experimentally validated BGCs [12,44]. Large-scale genomic repositories, including NCBI GenBank/RefSeq (https://www.ncbi.nlm.nih.gov, accessed on 1 August 2026), and JGI IMG/M (https://img.jgi.doe.gov, accessed on 1 August 2026), provide extensive datasets for mining and comparative analysis.

### 3.2. Resistance Gene Detection and Scoring

Following BGC annotation, candidate self-resistance genes are identified through an integrated strategy combining homology searches, essential gene analysis, gene duplication detection, and genomic proximity assessment. ARTS (https://arts.ziemertlab.com) provides a standardized framework for bacterial self-resistance genes systems [10,45]. Additionally, ARTS-DB (https://arts-db.ziemertlab.com) facilitates large-scale exploration of resistance–target relationships across bacterial genomes and metagenome-assembled genomes [46]. For fungal systems, FunARTS (https://funarts.ziemertlab.com) extends genome mining by integrating resistance gene information into BGC annotation, enabling target-directed analysis [11]. The Resistance Gene Database (RGDB) further supports annotation and large-scale screening by integrating multiple resistance-related datasets [17]. Candidate resistance genes are quantitatively evaluated using multi-parameter scoring systems, incorporating factors such as co-localization with BGCs, conservation across taxa, similarity to known resistance determinants, and mechanistic interpretability [17,37]. This scoring step is critical for distinguishing true self-resistance genes from unrelated genomic elements (Figure 3).

### 3.3. Target Inference and Cluster Prioritization

Candidate resistance determinants are then linked to potential molecular targets via conserved domain analysis, phylogenetic comparison with housekeeping homologs, and mapping to known drug targets. The genomic context of resistance genes further supports functional interpretation (Figure 3). For example, phylogenetic analysis has demonstrated the co-evolution of the odilorhabdin resistance gene oatA with its corresponding BGC [20]. Based on these analyses, BGCs are prioritized according to resistance signal strength, predicted novelty, taxonomic tractability, and feasibility of experimental activation or heterologous expression [17,47]. Gene Cluster Family (GCF) analysis is often applied to identify uncharacterized “orphan” clusters lacking known analogs [48]. This step is particularly powerful in identifying BGCs targeting eukaryotic systems, enabling the discovery of antifungal agents and herbicides with novel modes of action [24,47].

### 3.4. Experimental Validation

Computationally identified candidate BGCs require conversion into discrete compounds and mechanistic validation, including silent BGC activation, heterologous expression, fermentation optimization, metabolite isolation, resistance testing, gene knockout/overexpression, and biochemical/cellular target validation (Figure 3) [22,49,50,51,52]. Heterologous expression is critical for BGC function validation, with common hosts including *Aspergillus nidulans*, *Streptomyces species*, *Saccharomyces cerevisiae* and *Escherichia coli* [14,33,53,54]. And gene cluster capture techniques include yeast homologous recombination, TAR cloning, and CRISPR-Cas9 [55,56,57]. Cerulenin’s polyketide synthase gene cluster function was validated via heterologous reconstitution in *A. nidulans*, and the BGC of Fellutamide B was activated and its pathway elucidated via promoter exchange in *A. nidulans* [22,33]. Gene knockout and complementation experiments validate resistance gene function; for example, in vitro and in vivo experiments confirmed Cph phosphotransferase of capreomycin dual resistance mechanism [15], and functional verification of *Yersinia ruckeri*’s RNA methyltransferase self-resistance gene clarified its role in holomycin biosynthesis [8].

Fermentation products from heterologous expression systems or wild-type strains are isolated and purified, with structural characterization via HPLC-MS/MS and NMR spectroscopy. Structural elucidation of novel compounds lays the foundation for subsequent activity studies. This is a critical step in validation, with strategies including dual functional screening, crystal structure analysis, and in vitro/in vivo activity assays [16,21,38]. For streptocilpamides discovery, a dual-function platform combining fluorescence-based substrate hydrolysis and ADEP-induced ClpP activation antagonism screening identified novel ClpP inhibitors, with crystal structure analysis elucidating their mechanism [38]. FR901483’s self-resistance enzyme FrzK structural adaptability was revealed via crystal structure analysis [16]. Automation technologies have narrowed the gap between computational screening and laboratory validation, with the FAST-NPS platform as a representative fully automated, scalable system for *Streptomyces* bioactive natural product discovery—integrating self-resistance-guided screening with cloning, heterologous expression, fermentation, and product extraction, providing a path for large-scale high-throughput discovery [36].

## 4. Representative Advances

Self-resistance gene-guided discovery has achieved remarkable advances in both bacterial and fungal systems, expanding from classical antibacterial discovery to the mining of antifungals, herbicides, immunosuppressants, and antitumor agents. A 2020 NPR review listed many compound structures whose biosynthetic gene clusters harbor self-resistance genes [14], and the present review summarizes those discovered since 2020 (Table 1). This strategy has not only deepened mechanistic understanding of microbial self-protection but also facilitated the transition from fundamental research to scalable discovery pipelines and industrial application.

DNA replication is a fundamental process of life that requires the coordinated action of multiple enzyme complexes. Bacterial DNA replicase exhibits high evolutionary conservation with prokaryotes but significant divergence from eukaryotes, making it an ideal target for antimicrobial agents. In addition to the DNA replication inhibitors (Novobiocin, Chlorobiocin, Coumermycin, and Griselimycin) summarized in a 2020 review [14], Panter et al. had conducted systematic genome mining of myxobacteria using pentapeptide repeat proteins (PRPs) as probes—proteins previously identified as structural repeat elements (SREs) in the BGCs of gyrase inhibitors albicidin and cystobactamid. Through promoter engineering strategies to activate the PKS gene cluster containing a PRP-coding gene from *Pyxidicoccus fallax*, two novel compounds, pyxidicycline A and B (Table 1), were successfully produced. Bioactivity studies confirmed that both compounds act as selective topoisomerase inhibitors [58].

**Table 1:** Representative examples of BGCs employing self-resistance genes, with an emphasis on studies published between 2020 and 2026.

Natural Products	Source Organism	Function	Molecular Target	Biosynthetic Core Gene	Compounds Structures	Ref
1233A 1233B	*Fusarium* sp. RK97-94	Lipid metabolism inhibitor	HMG-CoA synthase	PKS	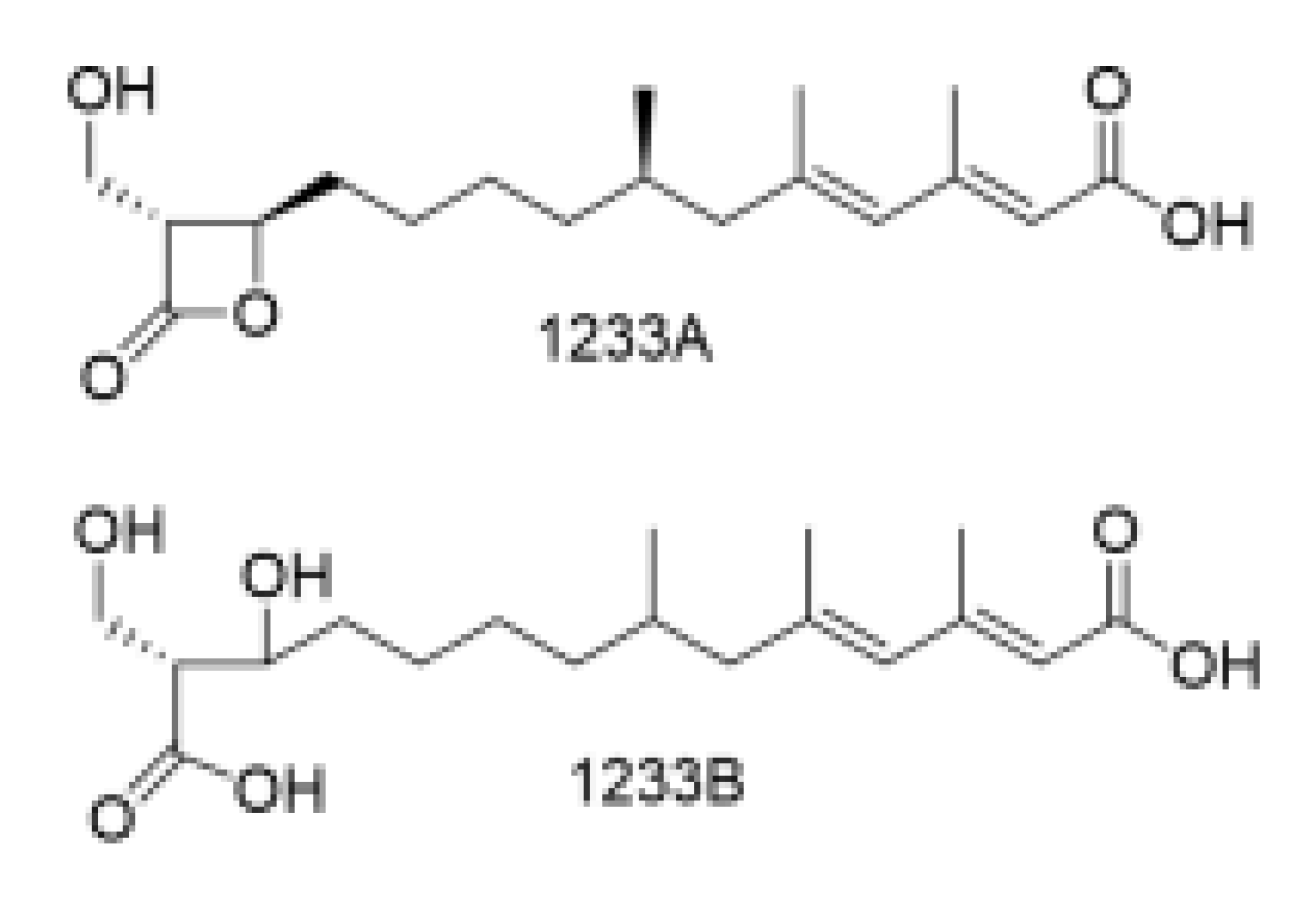	[59]
Acrophialocinol Acrophialocin	*Acrophialophora levis*	Other	Not specified (antifungal)	PKS	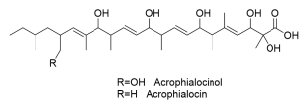	[9]
Alligamycin A/B	*Streptomyces iranensis*	Other	Fungal cell wall integrity	PKS	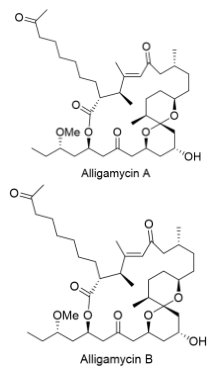	[42]
Aridomycin A/B	*Amycolatopsis* sp.	Lipid metabolism inhibitor	Not specified	PKS	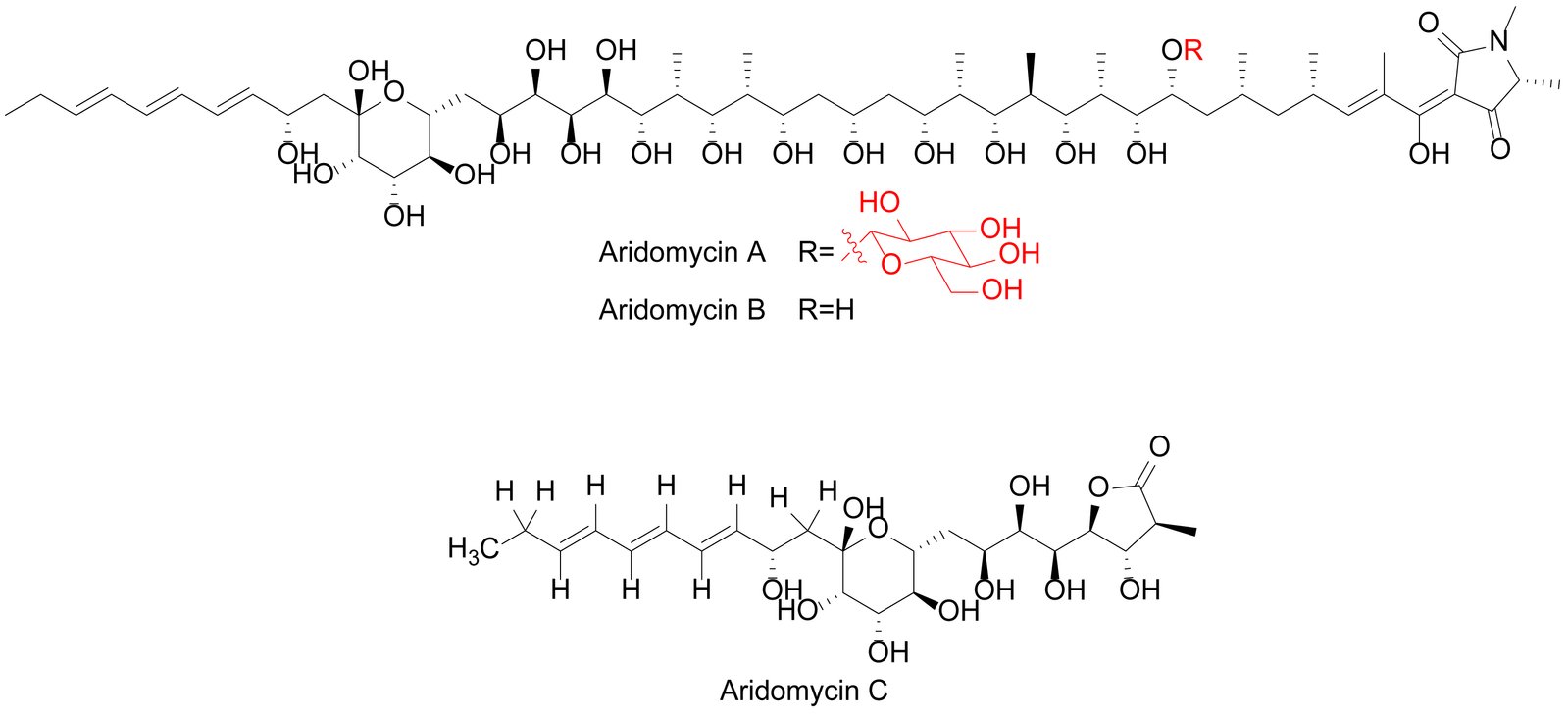	[60]
Capreomycin A/B	*Saccharothrix mutabilis* subsp. *capreolus*	Protein biosynthesis inhibitor	protein synthesis	NRPS	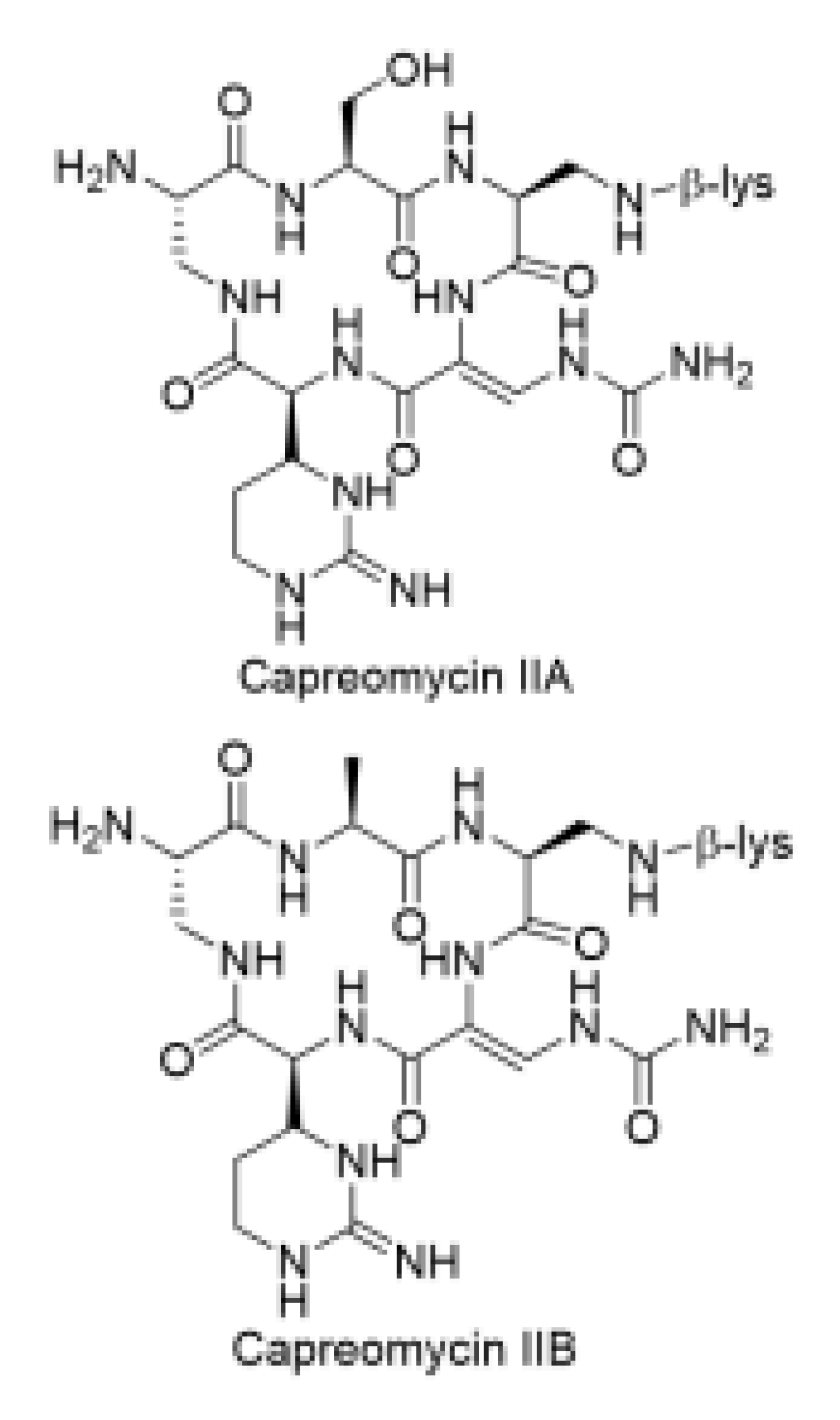	[15]
Cerulenin	*Sarocladium* spp.	Lipid metabolism inhibitor	FAS	PKS	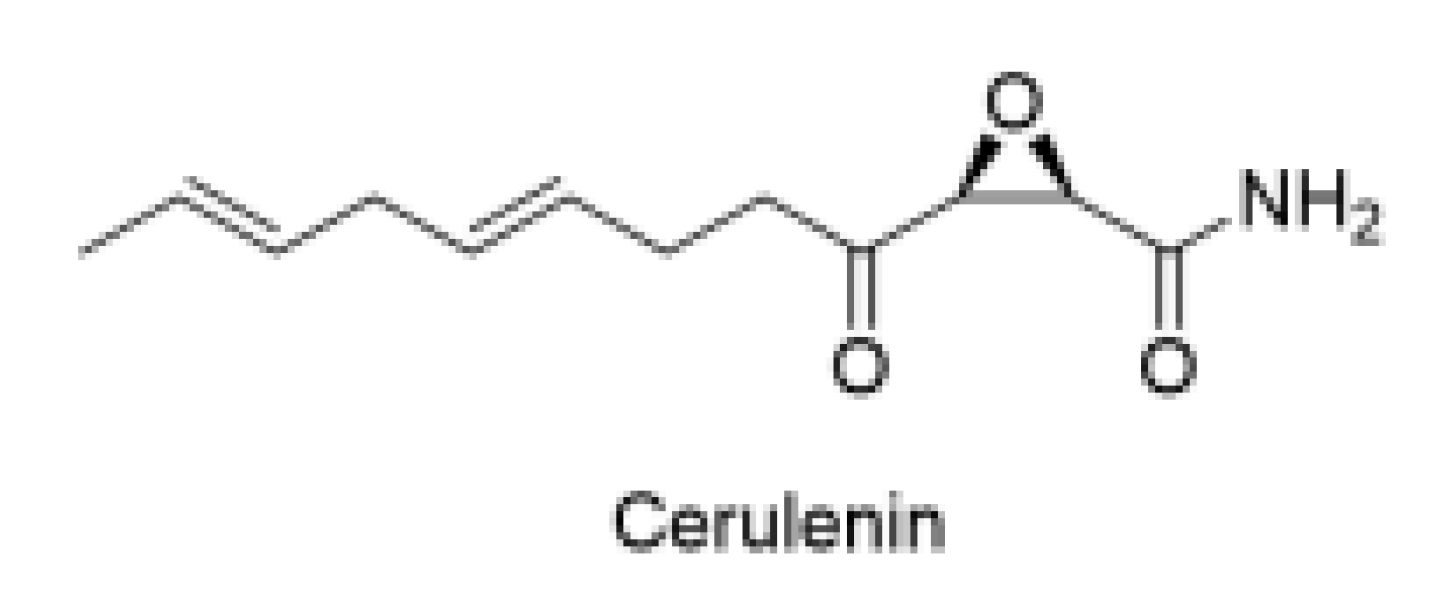	[22]
Cosmomycin D	*Streptomyces olindensis*	DNA metabolism inhibitor	DNA intercalation	PKS	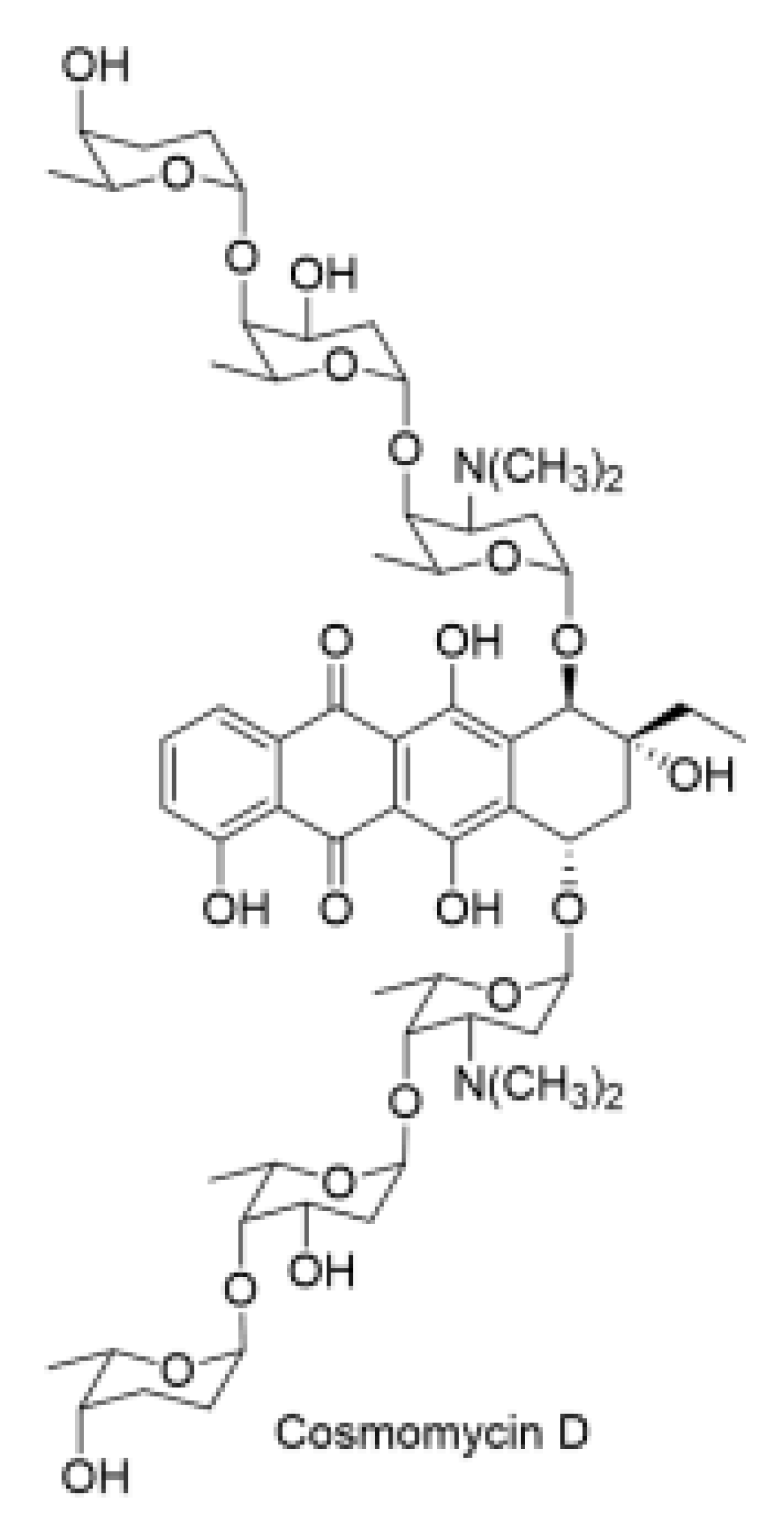	[39]
FR901483	*Cladobotryum* sp. No. 11231	Nucleotide metabolism inhibitor	PPAT	other	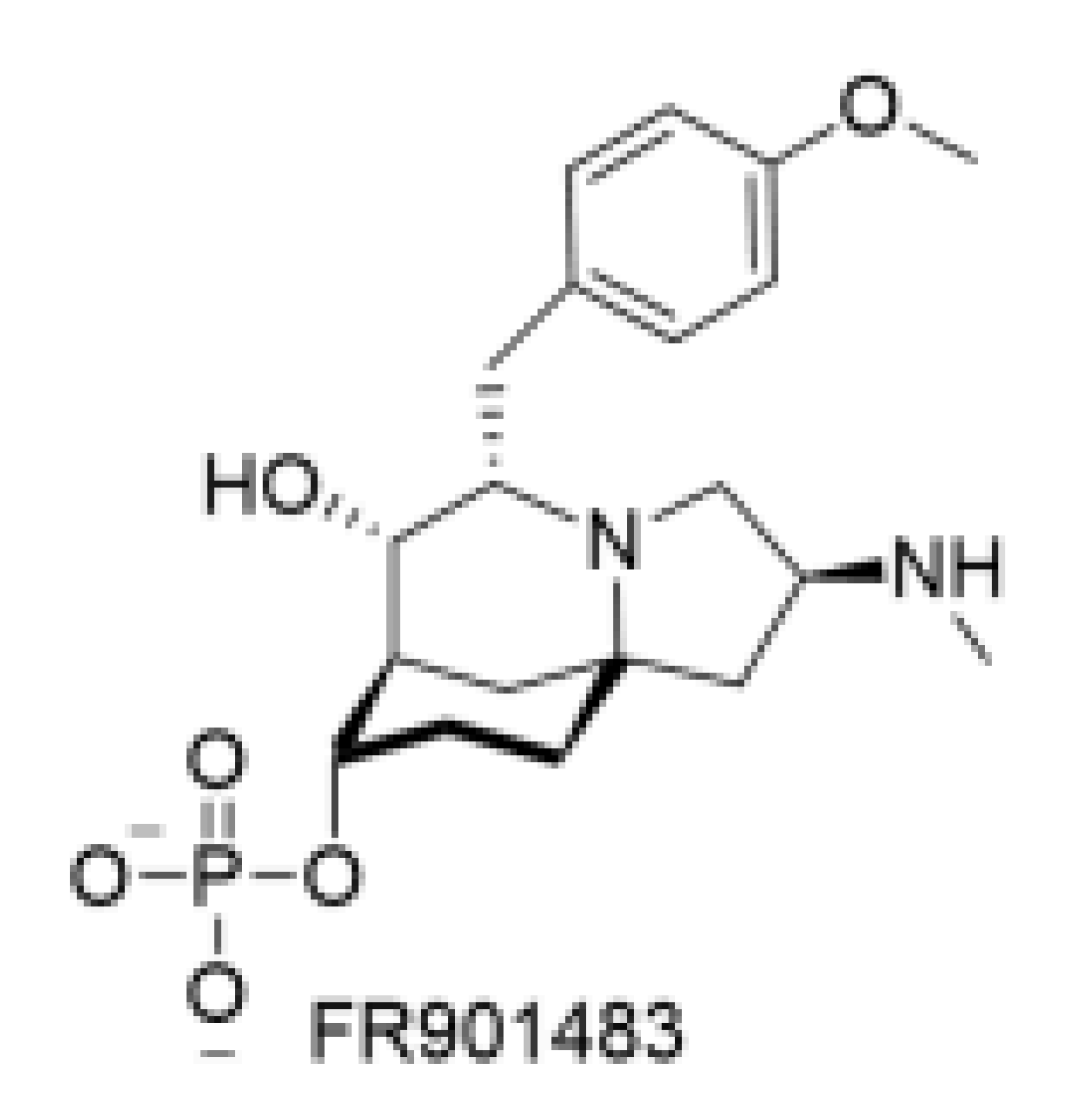	[16,61]
HB-35018	*Aspergillus terreus*	Amino acid metabolism inhibitor	ALS (Acetyl-Lactate Synthase)	PKS	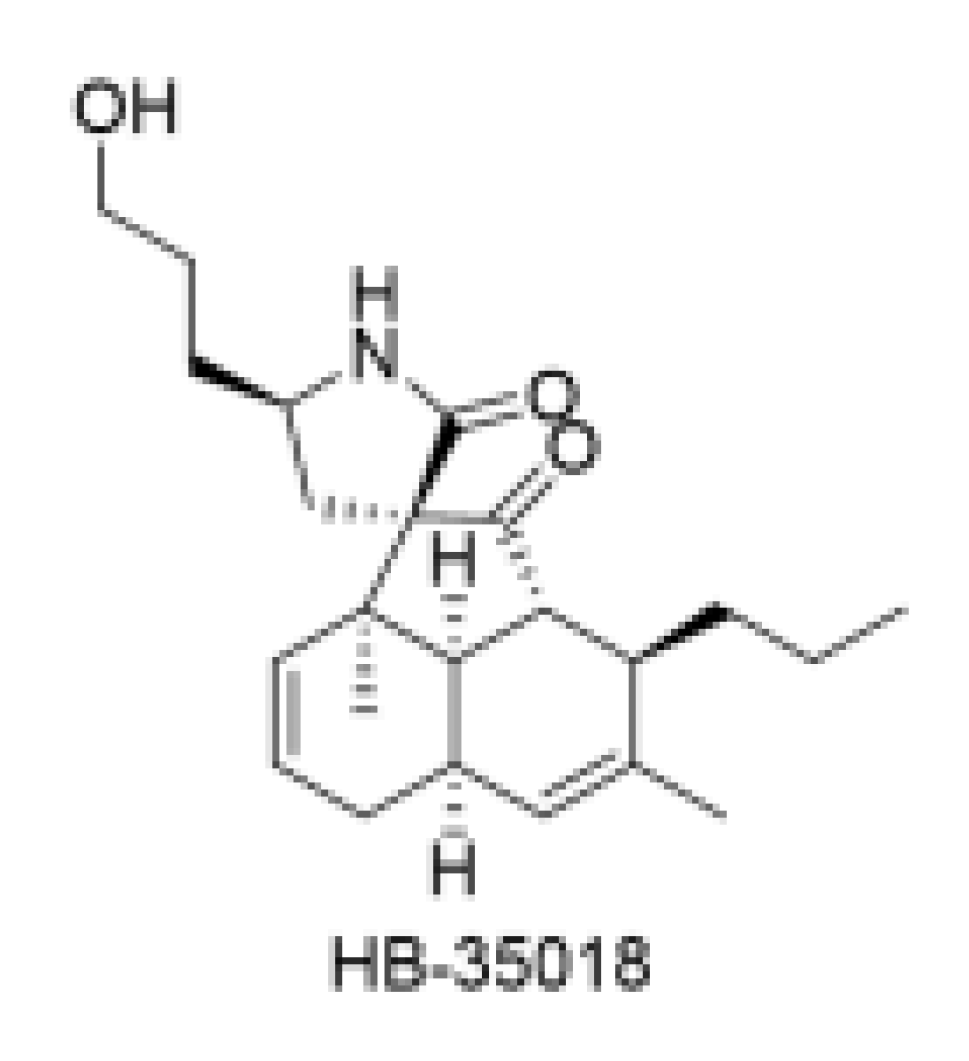	[24]
Mandimycin	*Kitasatospora melanogena*	Lipid metabolism inhibitor	Phospholipids in fungal cell membranes	PKS	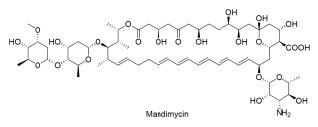	[41]
Myxin	*Lysobacter antibioticus* OH13	DNA metabolism inhibitor	Not specified	other	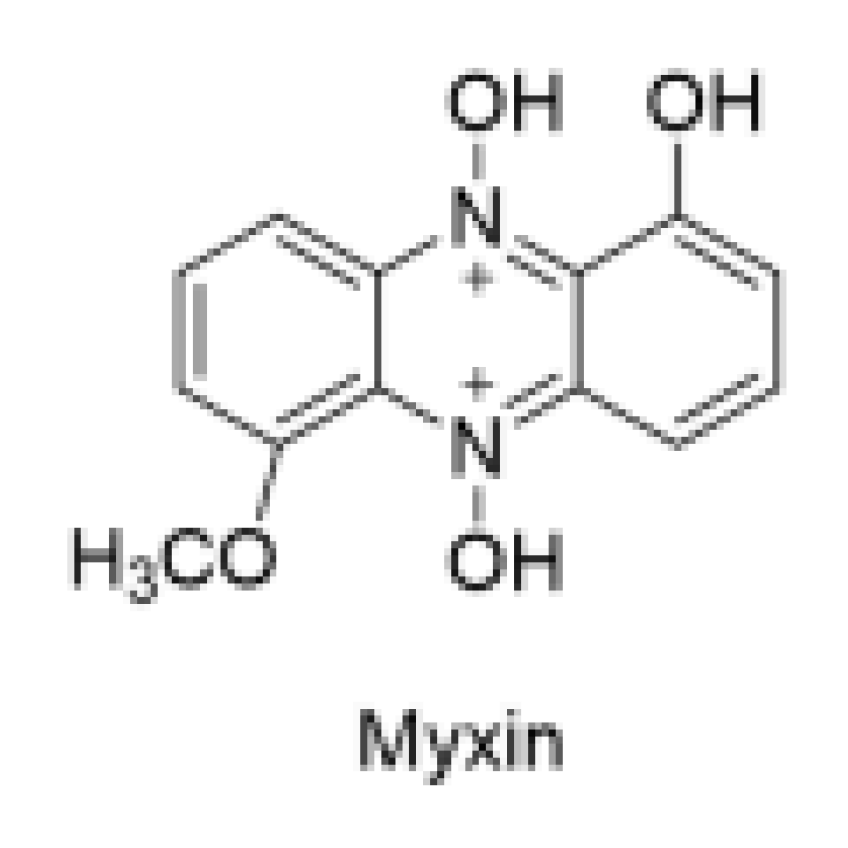	[40]
Pyxidicycline A/B	*Pyxidicoccus fallax*	DNA replication inhibitor	topoisomerase	PKS	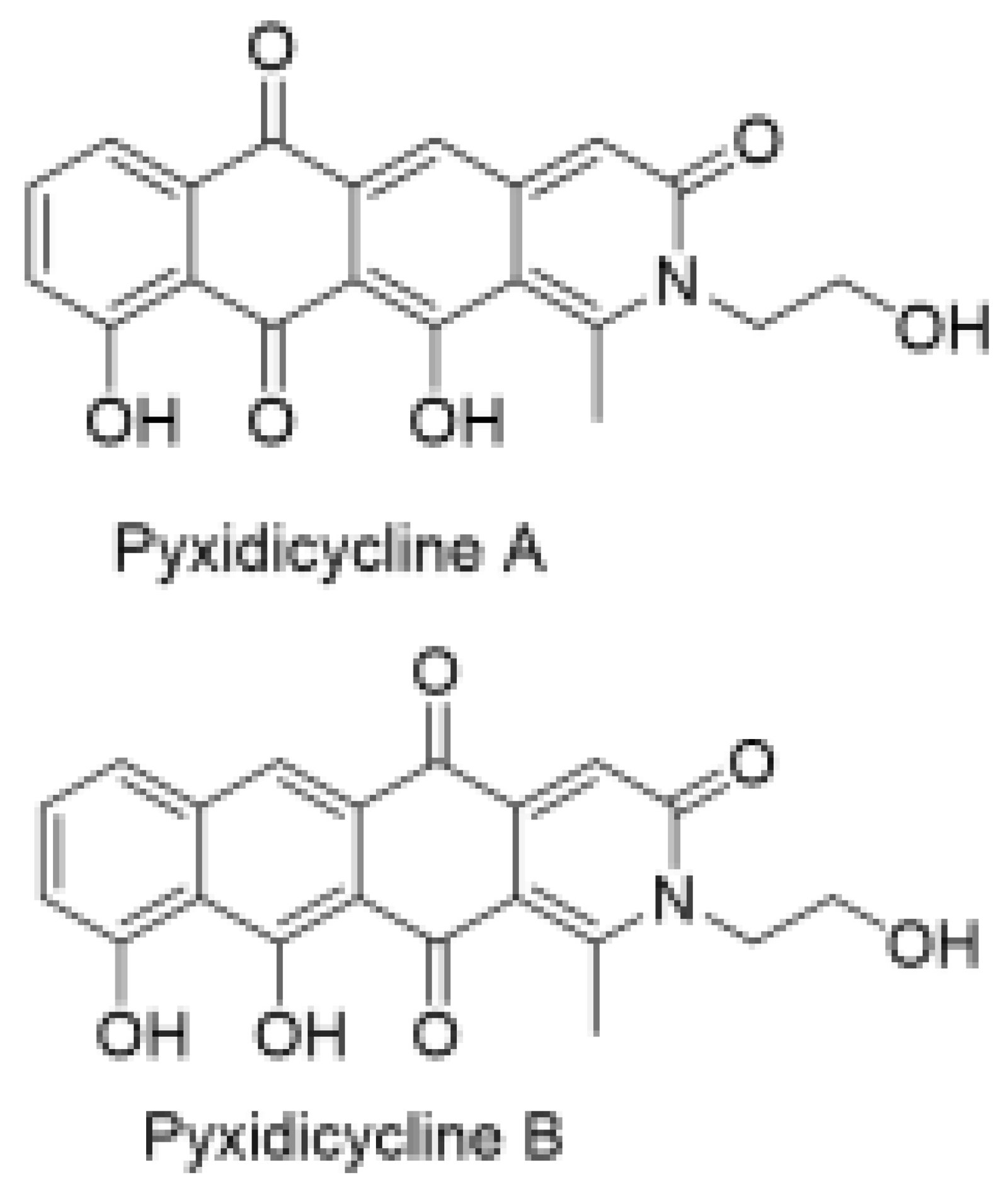	[58]
Restricticin Lanomycin	*Aspergillus nomius*	Lipid metabolism inhibitor	CYP51 (14α-demethylase of lanosterol)	PKS/NRPS	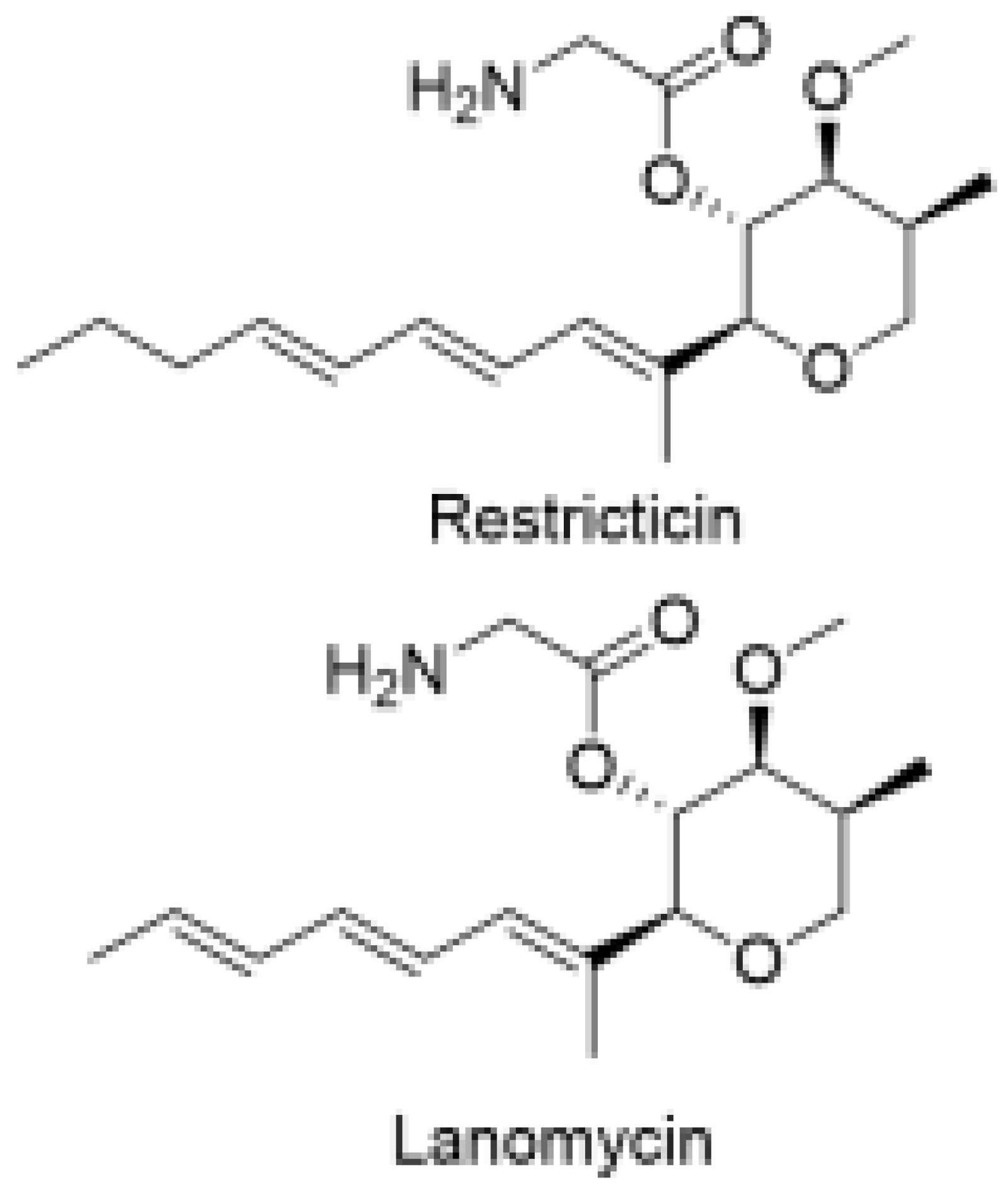	[34]
Streptoclipamide A	*Streptomyces* sp. S186	Protein degradation inhibitor	ClpP protease (antivirulence target)	NRPS	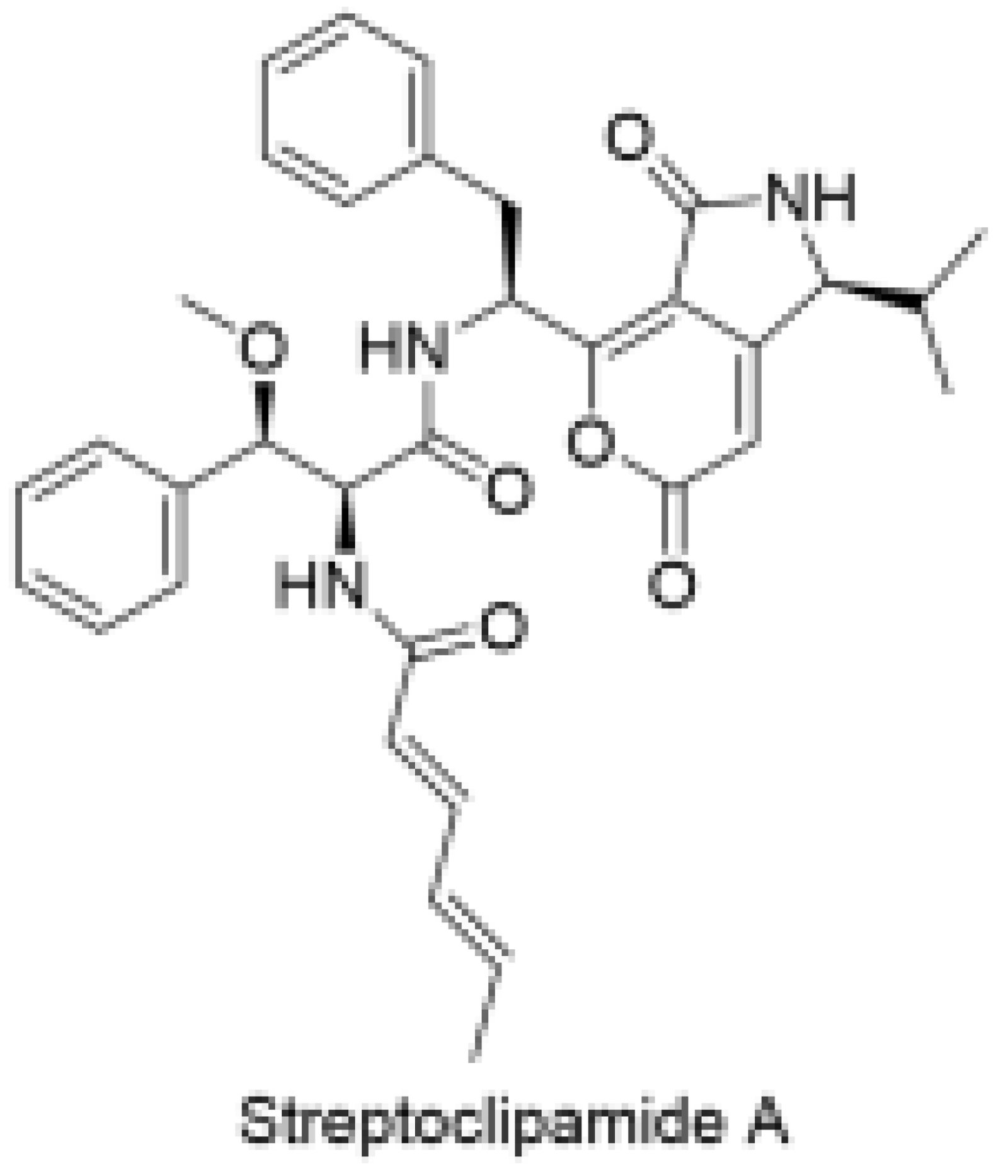	[38]
Vibriomycins A/B	*Vibrio ruber* DSM 16370	Protein biosynthesis inhibitor	Threonyl-tRNA synthetase (ThrRS, allosteric inhibitor)	PKS-NRPS hybridization	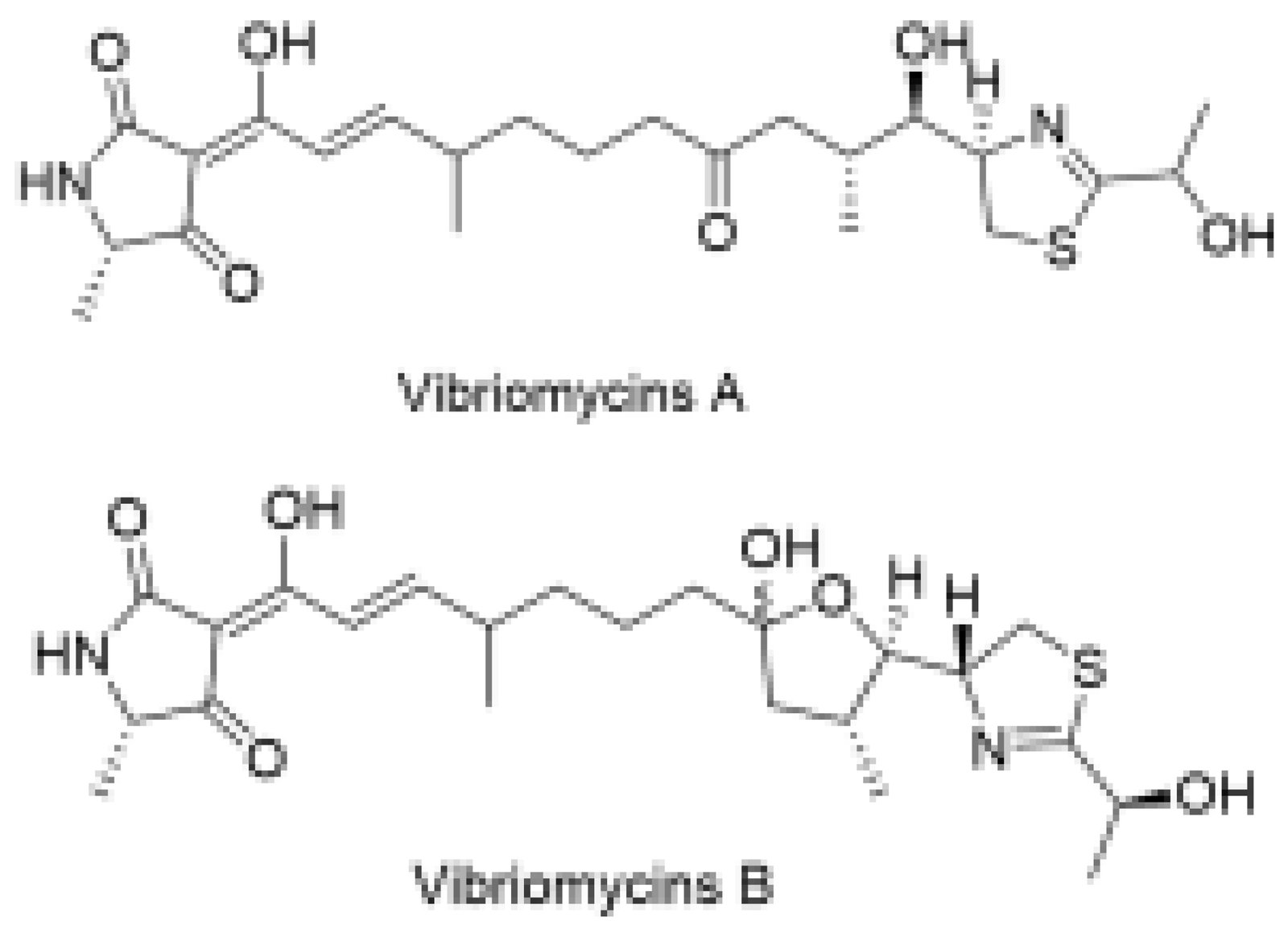	[21]

Protein biosynthesis is a key antimicrobial target due to significant differences between prokaryotic and eukaryotic systems. A previous review summarized various inhibitors and pathways targeting this process. Recently, Kishore et al. carried out self-resistance element (SRE)-guided genome mining on marine bacterium *Vibrio ruber* DSM 16370 by taking aminoacyl-tRNA synthetase (aaRS) homologs as probes [21]. The aaRS resistance gene co-localized with a PKS-NRPS hybrid BGC was identified via genomic analysis. Combined with gene mutagenesis and isotope labeling assays, three tautomeric congeners, vibriomycins A, B, and B’, were characterized as the main metabolites of this pathway. Biological assays verified that vibriomycins serve as novel allosteric inhibitors of bacterial threonyl-tRNA synthetase (ThrRS), which matches the target annotation in Table 1. Distinct from borrelidin and other ThrRS inhibitors with competitive binding modes, vibriomycins adopt an allosteric inhibitory mechanism, forming a new chemotype of antibacterial agents [21].

Proteasomes are key complexes controlling protein degradation in eukaryotic cells and serve as targets for antitumor drugs. The research team led by Fan Zhang recently developed an integrated discovery platform combining self-resistance gene-guided genomic mining with dual-function screening, which successfully identified anti-virulence inhibitors targeting ClpP proteases [38]. Using ClpP proteases as probes, they screened over 34,000 actinomycete genomes and identified 530 biosynthetic gene clusters (BGCs) containing potential ClpP self-resistance genes, with 26 selected for experimental validation. They adopted a dual-function screening strategy combining fluorescence detection and anti-ADEP-induced ClpP activation reverse screening. The platform successfully identified a series of ClpP inhibitors, including streptoclipamide A (IC_50_ = 480 nM). The BGC of streptoclipamide belongs to the NRPS type, encoding a mutated ClpP as a SRE. This represents the first reported natural product inhibitor targeting ClpP. Given that ClpP is a core factor regulating virulence in Methicillin-resistance *Staphylococcus aureu* (MRSA), this study not only provides promising lead compounds for anti-MRSA therapy but also establishes a scalable paradigm for natural product targeting screening [38].

DNA metabolism serves as a critical drug target. Castillo Arteaga et al. investigated the self-resistance mechanisms developed by *Streptomyces olindensis* DAUFPE 5622, a producer of the antitumor antibiotic cosmomycin D (COSD, an anthracycline family member) [39]. They identified or proposed three self-resistance mechanisms anchored in the COSD biosynthetic gene cluster: ABC transporter (cosIJ), UvrA class IIa protein (cosU), and a novel self-resistance mechanism mediated by mycothiol peroxidase (MPx) encoded by cosP. Activity studies of MPx and its mutants confirmed its involvement in peroxide-responsive mechanisms during COSD biosynthesis. Overexpression of ABC transporter, UvrA class IIa protein, and MPx all led to enhanced responses to toxic anthracyclines such as cosmycins. These findings indicate that cysteine peroxidase protects *S. olindensis* from peroxidative damage during COSD production, suggesting convergent resistance evolution between natural product-producing bacteria and tumor cells [39].

Sterols are essential components of eukaryotic cell membranes, and their biosynthetic pathways have long been exploited as targets for antifungal therapy. Among these, lanosterol 14α-demethylase (CYP51) is a well-established molecular target of azole antifungal agents and plays a central role in ergosterol biosynthesis. Natural products targeting CYP51 are relatively rare, with fungal metabolites such as restricticin representing one of the few known examples [62]. Leveraging this biological context, Liu et al. employed CYP51 as a self-resistance marker for target-directed genome mining, enabling the identification and experimental validation of the biosynthetic gene cluster associated with restricticin-related compounds [34]. Subsequent mining of biosynthetic gene clusters harboring CYP51 homologues further led to the discovery of lanomycin from previously uncharacterised fungal producers. These findings underscore the utility of self-resistance genes as functional indicators for linking biosynthetic gene clusters to their molecular targets, and highlight the potential of this strategy for uncovering bioactive natural products with defined modes of action.

About carbohydrates and energy metabolism inhibitors. Pentalenolactone and heptelidic acid both covalently inactivate glycerol-3-phosphate dehydrogenase (GAPDH) through their epoxy structures [63,64]. They all contain an additional GAPDH copy in their gene clusters and have been confirmed as autoresistant enzymes. Aurovertin E and citreoviridin target the ATP synthase β chain, with the mutant ATP synthase β chain encoded in their gene clusters conferring resistance to producing bacteria [65,66].

As a key enzyme in the branched-chain amino acid biosynthesis pathway, acetolactate synthase (ALS) is absent in humans, making it a potential antifungal target. Perlatti et al. first validated this concept by demonstrating the druggability of ALS. Subsequently, the team employed a resistance-gene-guided genomic mining strategy to identify a biosynthetic gene cluster from *A. terreus* that encodes HB-35018 [24]. This compound represents a new class of spiro-cis-decalin tetramic acids and displays potent ALS inhibition. Biochemical and antifungal assays revealed that HB-35018 outperforms known ALS inhibitors in efficacy against *A. fumigatus* and other pathogenic fungi. Cryo-electron microscopy structural studies uncovered a unique covalent binding mode between HB-35018 and ALS, distinct from those of previously reported inhibitors. Furthermore, the researchers confirmed that ALS is critical for fungal virulence in a murine model of invasive aspergillosis. Collectively, these findings establish ALS as a promising antifungal drug target and highlight the utility of resistance-gene-guided genomic mining in antifungal discovery [24].

For the nucleotide metabolism inhibitor, FR901483 is an immunosuppressant. It was isolated in 1996 from the fungus *Cladobotryum* sp. No. 11231. Its mechanism of action differs from that of FK506 and cyclosporine A. Instead of suppressing IL-2 production, it inhibits nucleic acid biosynthesis [67]. The biosynthetic pathway of FR901483 was subsequently elucidated. The *frz* gene cluster governs the production of FR901483. Within this cluster, *frz* encodes a homolog of phosphoribosyl pyrophosphate amidotransferase (PPAT). The key enzyme catalyzes the first committed step of de novo purine biosynthesis [68]. It was hypothesized that *frzK* encodes a PPAT variant capable of evading inhibition by FR901483. This variant would then serve as a self-resistance enzyme (SRE) for the producing strain. Recently, biochemical and structural analyses were conducted on FrzK together with its *Escherichia coli* homolog PurF. Recombinantly produced FrzK exhibited PPAT activity (though weaker than PurF), but it effectively evaded strong inhibition by FR901483. These results confirmed that the target of FR901483 is PPAT and that FrzK functions as an SRE by maintaining de novo purine biosynthetic capability in the presence of the inhibitor [16]. To elucidate the molecular basis of FrzK‘s evasive mechanism, the crystal structure of PurF in complex with FR901483 was determined. A homology model of FrzK was also constructed. Sequence and structural analyses revealed that residues unique to FrzK cluster near the Flexible Loop, which undergoes a disorder-to-order transition upon substrate binding. Kinetic studies of site-directed mutants further indicated how resistance may arise. FrzK appears to structurally predispose the Flexible Loop to adopt the active, closed conformation even when FR901483 is present.

Since 2020, natural products with novel mechanisms of action or unidentified targets have also been discovered. Wieder et al. identified two novel polyhydroxy polyketone compounds, acrophialocinol and acrophialocin, as major antifungal metabolites from *Acrophialophora levis* through bioactivity-guided isolation. Importantly, the study revealed that self-resistance to polyhydroxy polyketone compounds is mediated by a conserved RTA1-like protein encoded in the acr biosynthesis gene cluster. RTA1 family proteins are typically associated with sterol transport, marking the first reported involvement of such proteins in natural product self-resistance [9]. Deng et al. utilized a phylogeny-guided natural product discovery platform to report the discovery of a polyene antifungal antibiotic, mandimycin. Mandimycin is biosynthesized by the *mand* gene cluster, exhibiting an evolutionary pattern distinctly different from known polyene macrolide antibiotics and featuring three deoxy sugar modifications. The compound demonstrated potent and broad-spectrum bactericidal activity against multiple multidrug-resistant fungal pathogens in vitro and in vivo. Unlike known polyene macrolide antibiotics targeting ergosterol (e.g., amphotericin B), mandimycin possesses a unique mechanism of action—targeting multiple phospholipids in fungal cell membranes, leading to the efflux of essential ions from fungal cells. This multi-target binding capability endows it with robust bactericidal activity and resistance evasion potential. Although the compound was primarily discovered through a phylogeny-guided strategy rather than the classical SRE co-localization approach, the presence of resistance elements in its producing bacterial gene cluster and its distinctive mechanism of action provide novel directions for antifungal drug development [41].

## 5. Strengths and Limitations

Self-resistance gene-guided genome mining introduces a functional dimension into natural product discovery by prioritizing biosynthetic gene clusters (BGCs) that are more likely to encode bioactive molecules with defined targets, rather than treating all clusters equally [17,18]. Case studies of sulfonamide and azoxy antibiotic biosynthesis further validate the efficacy of this strategy in identifying novel BGCs with unique catalytic machineries [69,70].

A key advantage of this strategy is its ability to generate early mode-of-action hypotheses prior to compound isolation, thereby bridging the gap between sequence-based prediction and downstream functional characterization [14,17]. Self-resistance-guided mining has accelerated the discovery of novel antibiotics (e.g., pyranonigrin A from fungi, pyxidicyclines from myxobacteria) and enabled heterologous expression of cryptic clusters [58,71,72,73,74,75,76], while these natural products also show potential to overcome clinical drug resistance [28]. This approach is broadly applicable across bacterial and fungal systems and has expanded beyond antibacterial discovery to include herbicides, antifungals, and immunosuppressants, highlighting its versatility in medicine, agriculture, and chemical biology [9,24,47]. Moreover, the development of specialized bioinformatic tools (e.g., ARTS, FunARTS, antiSMASH) and curated databases (e.g., MIBiG, RGDB) has established a robust technical framework for standardized and high-throughput mining [11,17,37,77]. By focusing on BGCs associated with self-resistance genes—particularly those linked to essential housekeeping functions—this strategy effectively reduces rediscovery rates and significantly improves the likelihood of identifying novel compounds [8,21,22,38].

Despite these advantages, several limitations remain. False positives can arise because not all duplicated or BGC-adjacent genes function as true resistance determinants [18,23], while false negatives are common due to resistance mechanisms that rely on regulatory, physiological, or genome-dispersed features that are difficult to detect computationally [19,23,37]. Key limitations include misannotation of pseudogenes as functional resistance genes [78], technical hurdles in characterizing rare enzymatic reactions (e.g., N-S bond formation, tRNA-dependent transfer) [69,70,79], and the unculturability of many resistance-containing microbes [80], alongside variable gene patterns across microbial phyla [45,81]. In particular, complex self-resistance systems involving multiple enzymes, subcellular locations, or stage-specific functions remain challenging to predict [18]. The predictive power of resistance genes also varies, with duplicated housekeeping genes providing strong target clues, whereas general transporters often offer limited mechanistic insight [7,9,17]. Furthermore, experimental validation remains a major bottleneck, as silent BGCs, low heterologous expression efficiency, and difficulties in metabolite isolation can hinder confirmation of predicted functions [6,35,82]. In addition, current databases still lack comprehensive coverage of rare or unconventional resistance mechanisms, especially in underexplored microbial groups such as marine fungi and extremophiles [2,17]. Although numerous case studies support the practical value of resistance-guided genome mining, systematic quantitative benchmarking against untargeted genome mining strategies remains unavailable. Because published studies differ substantially in target classes, microbial taxa, screening workflows, and validation criteria, direct comparisons of discovery efficiency, hit rates, or false-discovery rates are currently difficult. Developing standardized benchmarking metrics will therefore be an important direction for future methodological evaluation. Therefore, the full potential of this strategy is best realized when integrated with biochemical validation, optimized expression systems, and emerging automation technologies [6,35,37].

## 6. Future Perspectives and Conclusions

Self-resistance gene-guided discovery has emerged as an effective function-oriented strategy in natural product research, linking biosynthetic gene clusters (BGCs) to biological targets prior to compound isolation and overcoming a key limitation of untargeted genome mining. Tools such as ARTS, antiSMASH, MIBiG, and FAST-NPS have made this approach increasingly scalable. Future progress will rely on integration with multi-omics, synthetic biology, automation, and artificial intelligence, enabling improved prediction models that incorporate sequence homology, synteny, domain architecture, and validated resistance–target relationships. Automated ‘foundry-style’ pipelines will facilitate high-throughput prioritization, cloning, expression, and metabolite characterization.

The strategy is expanding beyond antibacterials to eukaryotic-targeting compounds, including herbicides, antifungals, immunosuppressants, and metabolic regulators, while exploration of novel resistance mechanisms (e.g., spatiotemporal separation, membrane transport) and comprehensive resistance gene databases will further broaden its scope. Synthetic biology offers major opportunities—engineering artificial BGCs, optimizing heterologous expression, and reconstructing pathways to accelerate discovery and production. Despite its power, this approach is not a universal solution but a complementary enrichment tool best used alongside comparative genomics, metabolomics, and automation. Nevertheless, self-resistance genes serve as ‘functional beacons’ for discovering natural products with defined modes of action, positioning this strategy at the center of future bioactive compound development for medicine, agriculture, and beyond.

## Data Availability

No new data were created or analyzed in this study. Data sharing is not applicable to this article.
